# Quality Assessment Indicators for Well-Child Care in Primary Health Care: A Scoping Review of Global Trends, Standardization, and Dimensions of Care

**DOI:** 10.3390/children13030382

**Published:** 2026-03-09

**Authors:** Priscila Ribas de Farias Costa, Márcia Oliseski, Rita de Cássia Ribeiro-Silva, Ana Zaira da Silva, Rejane Queiroz, Carlos Lira, Izabele Lôbo, Elzo Pinto Júnior, Galba Freire Moita, Maria del Pilar Quispe, Maria Yury Ichihara, Rafael Barros, Carl Kendall, Ítalo Aguiar, Anya Vieira-Meyer, Rosa Livia Freitas de Almeida, Márcia Machado, Lígia Kerr

**Affiliations:** 1 School of Nutrition, Federal University of Bahia; Center of Data and Knowledge Integration for Health (CIDACS), Oswaldo Cruz Foundation, Salvador 40296-710, Brazil; rcrsilva@ufba.br; 2 Post-Graduation Program in Food, Nutrition and Health, Federal University of Bahia, Salvador 40110-909, Brazil; marciaoliseski@ufba.br (M.O.); izabele.lobo@ufba.br (I.L.); 3 Medical School Board, Federal University of Vale do São Francisco, Paulo Afonso 48605-780, Brazil; ana.zaira@univasf.edu.br; 4 Public Health Department, Federal University of Maranhão, São Luiz 65080-805, Brazil; queiroz.rejane@ufma.br; 5 Center of Data and Knowledge Integration for Health (CIDACS), Oswaldo Cruz Foundation, Salvador 40296-710, Brazil; carlos.rodrigo@ufba.br (C.L.); elzo.junior@fiocruz.br (E.P.J.); maria.yury@fiocruz.br (M.Y.I.); 6 Oswaldo Cruz Foundation, Eusébio 61773-270, Brazil; galba.moita@fiocruz.br (G.F.M.); anya@fiocruz.br (A.V.-M.); 7 Department for Health and Environmental Surveillance, Ministry of Health, Brasilia 70058-900, Brazil; maria.quispe@fiocruz.br; 8 Nurse School, Federal University of Bahia, Salvador 40170-110, Brazil; rafael.barros@ufba.br; 9 School of Public Health and Tropical Medicine, Tulane University, New Orleans, LA 70118, USA; carl.kendall@gmail.com; 10 Department of Community Health, Federal University of Ceará, Fortaleza 60020-181, Brazil; aguiar.iwo@gmail.com (Í.A.); rliviafa@gmail.com (R.L.F.d.A.); marciamachadoufc@gmail.com (M.M.); ligiakerr@gmail.com (L.K.)

**Keywords:** child health, comprehensive health care, family-centered care, health information systems, health services assessment, primary health care, quality indicators, well-child care

## Abstract

**Highlights:**

**What are the main findings?**

- Most studies assessing well-child care quality in Primary Health Care (PHC) use heterogeneous and non-standardized indicators, mainly focusing on structural and clinical aspects.
- Relational dimensions—such as communication, satisfaction, and family-centered care—are rarely included, limiting a comprehensive evaluation of service quality.

**What are the implications of the main findings?**

- There is an urgent need to develop and validate a standardized core set of comprehensive indicators that integrate structural, clinical, and relational components.
- Linking these indicators to national information systems (e.g., e-SUS) can strengthen evidence-based management and advance the monitoring of child health goals aligned with the 2030 Agenda for Sustainable Development.

**Abstract:**

**Background/Objectives:** Well-child care plays a critical role in promoting child health and monitoring growth and development within Primary Health Care (PHC), in line with international frameworks such as the WHO Global Strategy and the UN Sustainable Development Goals (SDGs). However, the absence of standardized quality indicators limits comparability across studies and hinders continuous improvement worldwide. This study aimed to map and analyze the indicators used to assess the quality of well-child care in global PHC settings. **Methods:** A scoping review was conducted following PRISMA-ScR and Joanna Briggs Institute methodological guidance, with a pre-registered protocol. Comprehensive searches were performed in May 2025 across fourteen databases and two gray literature sources, without language or time restrictions. Eligible studies assessed quality indicators for well-child care among children up to 5 years, 11 months, and 29 days. Two independent reviewers performed study selection and data extraction. **Results:** From 6052 records, 62 studies met inclusion criteria. Out of them, most (68%) used composite indicators, primarily from pre-existing tools (67%). While structural and clinical indicators—such as immunization and service accessibility—were predominant, there was a critical absence of relational indicators focusing on patient–provider interaction. This lack of standardization and neglect of the relational dimension significantly hinders international comparability and the assessment of family-centered care quality. **Conclusions:** Developing and validating a core set of standardized, comprehensive, and context-sensitive indicators integrating structural, clinical, and relational dimensions is essential. These should be linked to information systems to enable robust national and international comparison, strengthen evidence-based management, and drive continuous quality improvement to achieve the 2030 Agenda goals. These findings provide a foundation for policymakers to develop standardized monitoring tools that prioritize neglected relational aspects of care.

## 1. Introduction

Well-child care is a cornerstone of monitoring child growth and development, integrating health promotion activities within Primary Health Care (PHC) worldwide [1]. Internationally, these efforts are guided by the WHO Global Strategy for Women’s, Children’s and Adolescents’ Health (2016–2030) [2], which establishes three strategic objectives: Survive, Thrive, and Transform. This global framework focuses on ending preventable deaths, ensuring health and well-being, and expanding enabling environments through core priorities such as immunization, nutrition, developmental monitoring, and the strengthening of health systems [2].

These global priorities are reflected in various national health systems. In Brazil, for instance, well-child care is established as a comprehensive national endeavor within the Unified Health System (SUS). This model is characterized by intersectoral collaboration and guided by national policies, such as the National Policy for Comprehensive Child Health Care (PNAISC) [3], which align with the Sustainable Development Goals (SDGs) to reduce infant mortality and promote equitable care [4,5]. Within such health systems, well-child care is delivered through regular clinical visits where professionals assess vaccination status, growth parameters, and cognitive development, while providing essential guidance to caregivers [6].

The quality of well-child care services in PHC is directly linked to the effectiveness of care, significantly reducing hospitalizations for avoidable conditions and lowering neonatal and infant mortality [7,8]. However, a conceptual distinction is necessary: while evaluating the quality of care involves a multidimensional judgment of the health service’s ability to increase desired health outcomes, evaluating quality indicators focuses on the validity, reliability, and systematization of the specific metrics used to perform such measurement. To ensure excellence, the use of robust quality indicators is essential. These metrics allow for the measurement of implemented actions, identification of service gaps, and the standardization of best practices to guide more effective public policies [9,10].

Despite its recognized importance, a significant knowledge gap persists: there is a global lack of standardization regarding which indicators are most effective, particularly those capturing the relational dimensions of care. This absence of a core set of validated indicators hinders international comparisons and prevents the integration of health data into robust information systems [11,12]. Mapping these indicators is therefore essential not only to assess the service itself but to evaluate the adequacy of the metrics currently being employed.

In this context, this scoping review aimed to map the indicators available in the global literature for assessing the quality of well-child care services in PHC. Specifically, it seeks to answer the following research question: ‘What are the indicators currently utilized or proposed in the global literature to monitor and evaluate the quality of well-child care within primary health care settings?’ By synthesizing evidence from diverse health systems, this study provides a foundation for improving care practices and assists managers, professionals, and researchers in developing more effective and equitable monitoring tools.

## 2. Materials and Methods

This study is a scoping review conducted in accordance with the PRISMA Extension for Scoping Reviews (PRISMA-ScR) [13] and the Joanna Briggs Institute Manual for Evidence Synthesis [14]. The protocol was pre-registered on the Open Science Framework (doi:10.17605/OSF.IO/AJVRW).

Using the Population, Concept, and Context (PCC) framework—Population (children), Concept (well-child care indicators), and Context (PHC)—the following research question was formulated: What indicators are available to assess the quality of well-child care services provided in PHC?

### 2.1. Information Sources and Search Strategy

The search strategy was conducted in May 2025 and no filters or restrictions regarding the year of publication were applied during the search or the screening process. The search followed a three-step process, as recommended by Aromataris & Munn [14]. The first step involved an initial search limited to two online databases appropriate and relevant to the topic studied: Medline and Cumulative Index to Nursing and Allied Health Literature (CINAHL). This initial search was followed by an analysis of words in the titles and abstracts of retrieved articles, as well as the indexing terms used to describe them.

Next, two independent reviewers conducted the final search across the following databases: Medline (via PubMed), Web of Science, EMBASE, Latin American and Caribbean Health Sciences Literature (LILACS), CINAHL, and the Institutional Repository for Information Sharing (IRIS/WHO). For gray literature, the Brazilian Digital Library of Dissertations and Theses (BDTD) and Google Scholar were searched. Descriptors and their synonyms were selected from Medical Subject Headings (MeSH), Embase Subject Headings (Emtree), and Health Sciences Descriptors (DeCS). Free terms were also used when identified.

Finally, the reference lists of all included studies were screened to identify any additional relevant studies that were not captured in the initial search or indexed in the databases. Finally, authors of primary studies or reviews were contacted for additional relevant information.

The terms and their synonyms for the Population component were as follows: “Infant”, “Infant newborn”, “Child, Preschool”, “Preschool Child”, “Children, Preschool”, “Preschool Children”, “Pediatric Nursing”, “Nursings, Pediatric”, “Pediatric Nursings”, “Children and Young People’s Nursing”, and “Nursing, Pediatric.” For the Concept component: “Quality Indicators”, “Health Care”, “Quality Indicators, Healthcare”, “Healthcare Quality Indicator”, “Healthcare Quality Indicators”, “Indicator, Healthcare Quality”, “Indicators, Healthcare Quality”, “Quality Indicator, Healthcare”, “Health Metrics”, “Health Metric”, “Metrics, Health”, “Global Trigger Tool, Healthcare”, and “Healthcare Global Trigger Tool.” Finally, for the Context component: “Care, Primary Health”, “Health Care, Primary”, “Primary Healthcare”, “Healthcare, Primary”, “Primary Care”, “Care, Primary.” These term sets were combined using the Boolean operators “AND” and “OR” and applied to all databases.

No restrictions on language, publication year, or study location were applied during the search and selection process.

Search strings are available in full as Supplementary Material (Table S1).

### 2.2. Eligibility Criteria

Eligibility criteria were defined using the PCC framework [14]. Studies were included if they met the following criteria: [i] evaluated quality indicators for well-child care services in a primary health care setting, [ii] involved a study population of children up to 5 years, 11 months, and 29 days of age, and [iii] were experimental or observational studies [cross-sectional, ecological, case–control, and cohort], systematic reviews, meta-analyses, conference abstracts, theses, or dissertations.

The exclusion criteria were as follows: [i] studies not conducted in a PHC setting, [ii] studies involving children older than 5 years, 11 months, and 29 days, and [iii] case reports, narrative reviews, short communications, editorials, and study protocols.

### 2.3. Study Selection and Data Extraction

The results from the database searches were exported to the web version of Endnote^®^, where duplicates were removed. The results were then imported into Rayyan^®^, where two independent reviewers selected the studies. The first step involved screening titles and abstracts; any disagreements were resolved by consensus or through consultation with a third reviewer. Subsequently, the second stage of selection involved a full-text review, also conducted independently by the two reviewers; any disagreements were resolved by consensus or by a third reviewer.

To ensure the rigor and reliability of the data synthesis, data were extracted by one reviewer, and the accuracy of the collected information was confirmed by the second reviewer. Disagreements regarding the extracted data were discussed and resolved by a third reviewer. Furthermore, the research team engaged in constant reflexivity, maintaining a neutral analytical position to ensure that the mapping of child health indicators remained grounded in the evidence, independent of the researchers’ prior professional expertise in specific health systems. Data were extracted into a Microsoft Office^®^ Excel spreadsheet. The information collected included: title, authors, publication date, study location, study design, sample size and participant characteristics [where available], methodologies for indicator development, statistical techniques used, data used for indicator construction, indicator performance, other relevant characteristics of the indicators, and the main study findings. Authors were contacted for missing information using the email address provided in the publication.

### 2.4. Data Synthesis

The collected data are presented descriptively in tables, figures, and graphs. The results are also mapped according to geographical regions and the concepts employed. A descriptive qualitative content analysis was conducted based on the thematic analysis approach outlined by Braun and Clarke [15]. This process was operationalized through a directed content analysis, using pre-defined categories such as the type and amount of information included in the indicators, the statistical techniques employed, and indicator performance. Quantitative data were synthesized using simple and absolute frequencies.

### 2.5. Ethical Considerations

Since this study consists of a scoping review of publicly available secondary data, it did not require formal approval from an Institutional Review Board (IRB). However, the review adhered to ethical principles of research integrity, ensuring the accuracy of data extraction, the transparent reporting of results, and the proper attribution of authorship for all included studies.

## 3. Results

### 3.1. Selection Process for Sources of Evidence

**Figure 1:**
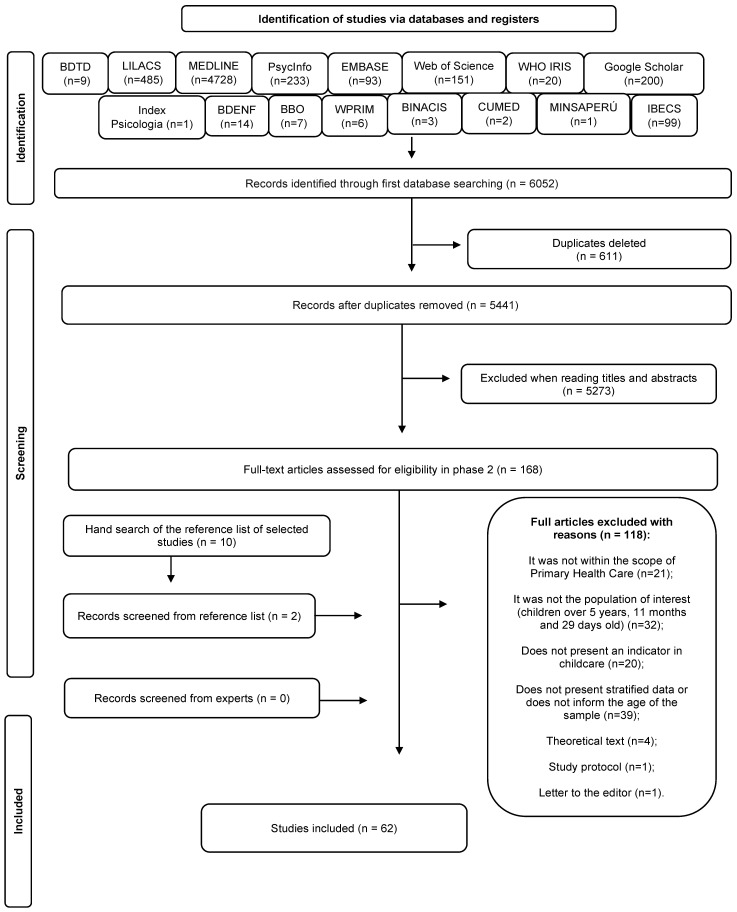
Flowchart of the study selection process.

The initial search across fourteen databases and two gray literature sources yielded 6052 records, which were reduced to 5441 unique citations after deduplication. Following the title and abstract assessment, 5273 records were excluded, leaving 168 articles for full-text eligibility assessment. To ensure the comprehensiveness of this review, manual searches and reference chaining were performed, contributing an additional 12 studies to the 168 identified via database searching, totaling 180 articles for full-text review. After a rigorous full-text eligibility assessment against pre-defined criteria, 62 studies [16,17,18,19,20,21,22,23,24,25,26,27,28,29,30,31,32,33,34,35,36,37,38,39,40,41,42,43,44,45,46,47,48,49,50,51,52,53,54,55,56,57,58,59,60,61,62,63,64,65,66,67,68,69,70,71,72,73,74,75,76] were ultimately selected for inclusion (Figure 1). The substantial reduction from the initial identification to the final inclusion—driven largely by the exclusion of over 5000 records during title/abstract screening—underscores a high sensitivity in the search strategy.

### 3.2. Characteristics of Included Studies

**Figure 2:**
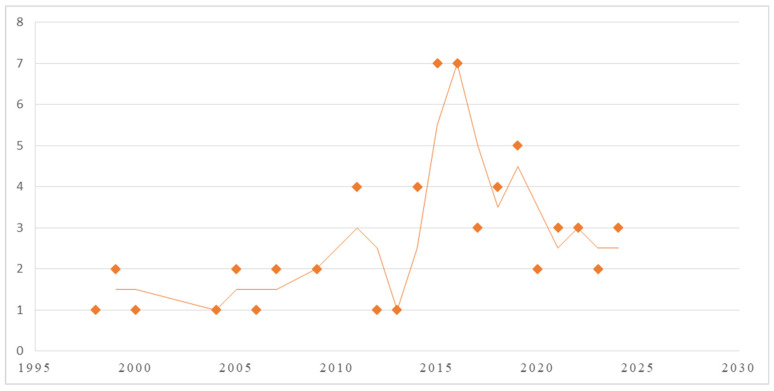
Publication period of the articles.

The temporal distribution of the literature spans over three decades (1993–2024), with a peak in publication volume occurring in 2015 (Figure 2).

Methodologically, the body of evidence is predominantly comprised of observational and methodological designs, which account for 92% (n = 57) of the included sources. Cross-sectional studies represent the most frequent design, followed by cohort and validation studies. Other approaches, including ecological studies, index development, and consensus methodologies, were less frequent, reflecting a diverse but primarily descriptive landscape of evidence (Table S2).

Formal interventional designs, such as clinical trials or quasi-experimental studies, represented a minority of the sample (8%; n = 5). Among them, distinct strategies were employed to improve healthcare quality. These included a cluster randomized trial evaluating a pay-for-performance (P4P) scheme through financial incentives in primary care facilities; a quasi-experimental study implementing an ‘Enhancing Healthcare’ (EHC) package focused on the ‘Find, Connect, Treat, and Retain’ strategy; a community-based before-and-after study aimed at optimizing the clinical management of acute bronchiolitis epidemics; a Quality Improvement (QI) collaborative based on the Breakthrough Series (BTS) model, utilizing peer-to-peer learning and structured mentoring visits; and a time series analysis (quasi-experimental approach) to evaluate the effects of extreme weather events on women’s and children’s access to essential health services (Table S2). These interventions were primarily designed to strengthen service delivery, enhance adherence to clinical protocols, and improve maternal and child health outcomes through data-driven performance monitoring.

**Figure 3:**
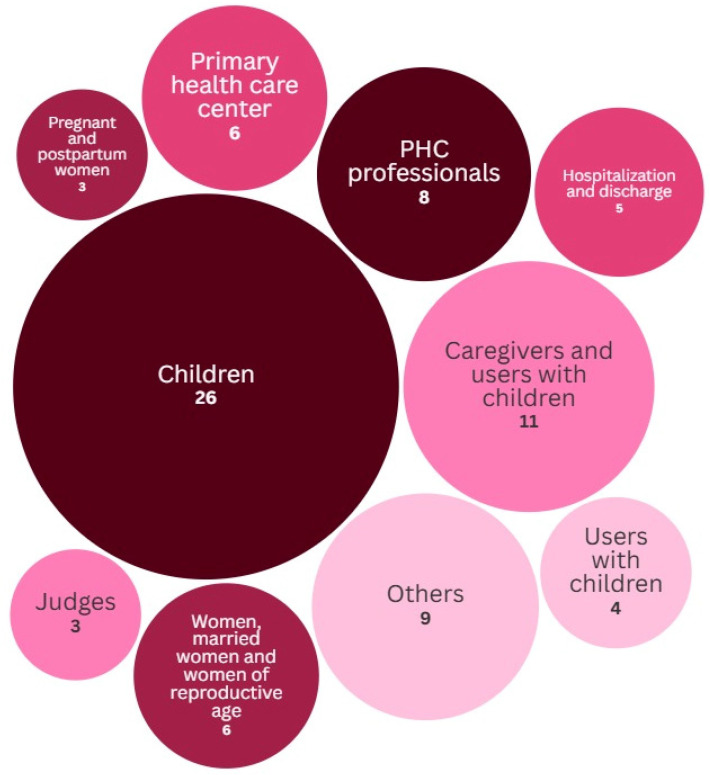
Context and Population of the Included Studies.

The study contexts varied. Six were conducted in PHC centers, eight involved PHC professionals, and 26 focused on children under 5 years of age. Three studies assessed pregnant and postpartum women, while one evaluated only children with bronchitis episodes. Another three were conducted with child health care experts/judges, and five assessed the number of hospitalizations and discharges. Eleven studies involved caregivers and users of PHC services for children, four included only PHC users with children, and six analyzed women of reproductive age. The remaining nine studies analyzed various indicators, such as medication prescriptions, patient residences, and bronchitis episodes (Figure 3). Among the studies that reported participant gender, 5 were conducted exclusively with women, and 19 included both men and women. The diversity of contexts reflects a broad but fragmented application of indicators across different PHC dimensions. Notably, the heavy concentration on clinical and demographic subgroups (e.g., children under five and hospitalizations) contrasts with the limited focus on the interaction between caregivers and professionals.

**Figure 4:**
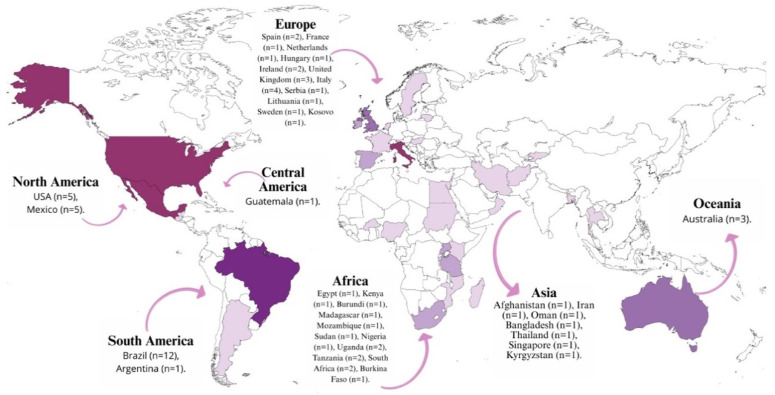
Geographical Distribution of Studies Included in This Review.

The review identified a wide geographic spread across 62 studies. Brazil was the most frequent study location (n = 11), followed by a significant representation from North America (USA and Mexico, n = 5 each) and Europe (Italy, n = 4; UK, n = 3; Ireland and Spain, n = 2 each). The Oceanian and African continents were also represented through studies in Australia (n = 3), South Africa, Tanzania, and Uganda (n = 2 each). The remaining 24 publications were distributed across 24 different countries (Figure 4), underscoring the global effort to establish quality indicators for well-child care.

### 3.3. Quality Assessment Indicators for Well-Child Care

**Figure 5:**
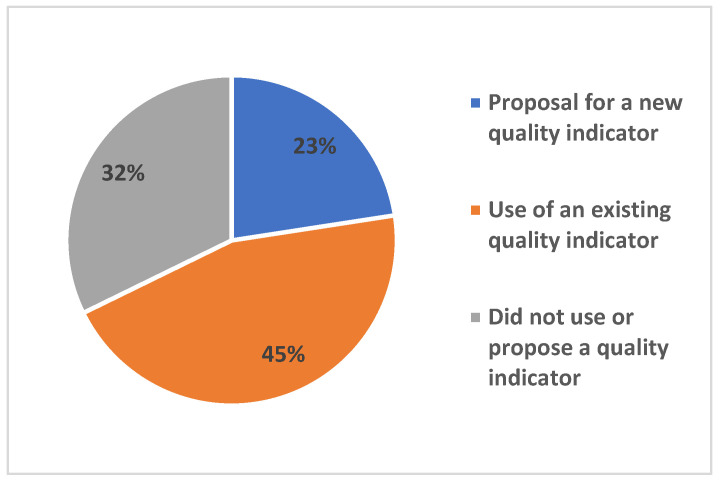
Proportion of Studies by Use of Composite Quality Assessment Indicators for Well-Child Care Services.

In this review, 20 studies (32%) did not use a composite indicator to assess the quality of child health care services, whereas the majority (68%; n = 42) did. Of these, 28 (67%) used a pre-existing quality indicator, while 14 (33%) developed a new indicator for quality assessment (Figure 5). Among the studies that developed a new indicator, 10 validated the instrument using analyses such as the Delphi method, principal component analysis, area under the ROC curve, and the Kappa statistic. These showed varied performance, ranging from “not specified” to “very good” (Table S1). The reliance on pre-existing indicators suggests a degree of consolidation in child health metrics, yet the development of new tools indicates ongoing efforts to address specific gaps. However, the predominance of quantitative validation (e.g., PCA and ROC) often prioritizes clinical and structural variables. This technical focus may explain the low presence of relational indicators identified in this review, as interpersonal and longitudinal dimensions of care are inherently more complex to psychometrically validate compared to discrete clinical outcomes.

In Table 1, the synthesized evidence was categorized according to the multi-dimensional model for PHC quality assessment proposed by Martufi et al. (2025) [77]. This framework organizes indicators into two primary clusters: a Structural Block, encompassing five components related to infrastructure, supplies, referral systems, work processes, and workforce quality; and a Service Cluster Block, specifically focused on Maternal and Child Health (MCH) services, including prenatal care, child care, and immunization practices (Table 1).

The mapping of indicators revealed a predominant focus on the Structural Block, as defined by Martufi et al. [77], with 93.5% of the studies addressing components such as workforce quality and the planning of service delivery. Within this block, the evidence was evenly distributed across infrastructure, general supplies, and referral systems, reflecting a global concern with the material and organizational foundations of Primary Health Care (PHC).

Regarding the Service Cluster Block for Maternal and Child Health, more than half of the relevant studies (54.7%) prioritized the ‘Availability and quality of child care’ component. This underscores a robust scientific interest in the technical delivery of pediatric services, although prenatal care and immunization practices also emerged as recurring themes (Table 1).

Interestingly, a subset of the literature occupied a space outside these established blocks. These studies were primarily focused on the methodological development of new indicators or on capturing the perceptions of caregivers and healthcare professionals. The presence of these studies highlights a growing, yet still marginalized, trend toward incorporating subjective and relational dimensions into quality monitoring—aspects that are often less represented in traditional structural and service-oriented frameworks.

**Figure 6:**
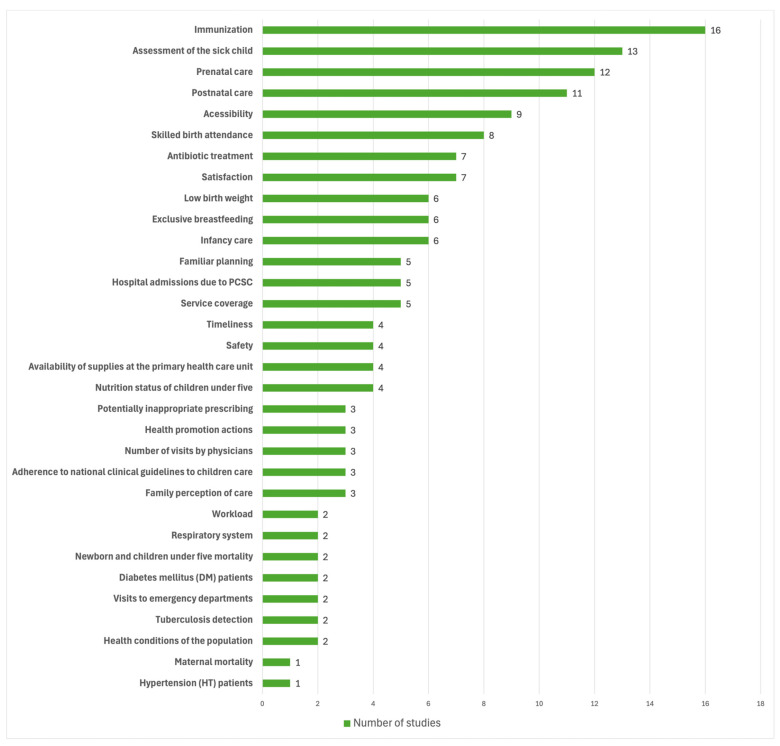
Main Quality Assessment Components for Well-Child Care in PHC.

The analysis of individual components reveals a predominant focus on routine clinical processes and access metrics. Immunization (n = 16) and sick child care (n = 13) emerged as the most frequent indicators, followed by prenatal and postnatal care (Figure 6). Aspects of healthcare access and skilled birth attendance were also moderately represented. In contrast, outcome-oriented indicators—specifically neonatal, infant, and maternal mortality rates—were markedly underrepresented, appearing in only one or two of the sixty-two studies (Figure 6). Other fundamental indicators of child health, such as exclusive breastfeeding and low birth weight, showed intermediate frequency (six studies each).

## 4. Discussion

The findings of this review highlight the global prioritization of indicators focused on child growth and development, vaccination coverage, and the strengthening of the therapeutic bond in PHC, aligning with international benchmarks, including the WHO’s Global Strategy for Women’s, Children’s and Adolescents’ Health [8] and the UN’s 2030 Agenda for Sustainable Development [78]. These data demonstrate that healthcare monitoring remains predominantly centered on health promotion and illness prevention through established clinical metrics. However, this evidence also reveals a widespread lack of uniformity in indicator definitions, which directly compromises data comparability across diverse socioeconomic contexts. This lack of standardization is not merely a technical flaw but reflects a global fragmentation of health services and local protocols [11]. Consequently, while the field is well-mapped descriptively through the identified cross-sectional studies, there is a clear evidence gap regarding longitudinal or experimental research capable of establishing causal links or assessing long-term program impacts.

A significant challenge identified through our evidence synthesis is the persistent disconnection between research findings and national Health Information Systems (HIS). The data obtained in this review indicate that while digital platforms for data entry and real-time monitoring have been widely developed globally, these tools remain chronically underutilized for strategic evaluation. This underutilization represents a critical evidentiary gap that undermines the longitudinal tracking of child health and obscures the early detection of systemic inequalities [79,80]. To address this, it is essential that health authorities move beyond the mere implementation of digital infrastructure, prioritizing the integration of research-validated metrics into routine HIS to transition from passive data collection to active, evidence-based management.

Furthermore, our findings regarding the scarcity of standardized relational indicators provide empirical support for concerns raised by international bodies. The evidence shows a clear asymmetry: while clinical outcomes such as immunization are rigorously monitored, metrics for the quality of provider-family interaction remain underdeveloped and fragmented. This observation aligns with reports from the OECD [81] and the WHO Nurturing Care Framework [82], suggesting that the current monitoring model is insufficient to capture the patient’s perspective [83]. In light of this, it is imperative that future PHC frameworks institutionalize relational indicators as core quality components. Bridging this gap requires not only the development of new metrics but also a shift in information system design to accommodate the subjective dimensions of care, ensuring a truly comprehensive and person-centered monitoring approach.

Identifying and analyzing indicators for well-child care are crucial steps in understanding how care is structured and delivered within the public health system. Beyond simply measuring performance, these indicators allow for assessing the effectiveness of interventions, guiding resource allocation, and informing strategic decisions in PHC management [84,85]. However, we observed that while clinical indicators [such as immunization and anthropometry] are well-established, there is a significant gap in measuring the subjective and relational dimensions of care.

Quality in well-child care extends beyond technical aspects to include the healthcare team’s ability to build trusting relationships and communicate effectively with families. Factors such as active listening, empathy, a welcoming environment, and the user’s perception of the service are crucial for adherence to preventive practices, including breastfeeding, vaccination schedules, and regular check-ups [11,85]. Actively listening to users enables the identification of expectations and needs that traditional indicators do not capture, thereby strengthening the patient–provider bond and enhancing the care experience [86,87]. However, this study shows that these aspects are rarely incorporated into formal assessments, representing a critical area for improvement.

In light of these gaps, it is imperative to develop a minimum set of comprehensive, sensitive, and specific indicators for well-child care in PHC. This set of indicators should encompass not only clinical dimensions—such as growth, development, and immunization—but also structural [e.g., infrastructure, availability of supplies, service organization] and relational dimensions [e.g., quality of the patient–provider bond, user satisfaction, and experience] [79,84]. To be effective, these indicators must be based on robust scientific evidence, aligned with national and international public policies, integrated into health information systems, and validated across diverse contexts [14,78]. Validation is a crucial step, as it ensures that the indicators accurately reflect the realities of different regions and are comparable on a national level. Furthermore, their adoption must be accompanied by a continuous process of monitoring and feedback, enabling managers and healthcare teams to rapidly identify problem areas and implement corrective measures [14].

Future research should focus on developing and applying indicators that incorporate not only objective performance measures but also variables related to user satisfaction and experience, thereby providing a more holistic, person-centered view of the quality of care [86,87]. This broader approach can help build a model of well-child care that is more effective, equitable, and responsive to the actual needs of children and their families.

Major strengths of this review include its rigorous methodology following JBI recommendations and a comprehensive search across fourteen databases without language or time restrictions. This approach ensured a representative global sample. Such diversity reinforces the validity of our findings regarding the global lack of standardized relational indicators. Despite the rigorous methodology, this review has limitations. Although no language restrictions were applied, the predominance of studies in English, Portuguese, and Spanish may have limited the inclusion of relevant evidence from other regions. Furthermore, the high degree of methodological and conceptual heterogeneity among the included studies represents a significant challenge. The diversity in how quality indicators are defined, operationalized, and measured across different health systems hindered direct comparisons between findings. This fragmentation underscores the nascent stage of standardization in child wellness metrics and implies that the synthesized results should be interpreted as a qualitative landscape of the field rather than a uniform global baseline.

Our evidence suggests that PHC managers should look beyond clinical outcomes to incorporate relational metrics—such as caregiver satisfaction and the quality of the provider-family bond—into formal monitoring systems. Such a shift is not merely conceptual but operational; the integration of these indicators into digital Health Information Systems (HIS) is essential for enabling real-time, evidence-based management. By leveraging digital health infrastructure to capture the interpersonal dimensions of care, health systems can better identify service gaps and proactively reduce health inequalities in child follow-up, ensuring that the continuity of care is both clinically robust and humanized.

The findings provide a foundation for improving clinical practices and management strategies worldwide. Standardizing indicators can support the development of monitoring protocols integrated with digital health systems, strengthening evidence-based management [84]. In clinical practice, prioritizing the user experience can improve family adherence, enhancing continuity of care. Ultimately, adopting a standardized minimum set of indicators would promote greater global equity, ensuring child health targets are monitored consistently in alignment with the 2030 Agenda [78].

## 5. Conclusions

In conclusion, this scoping review establishes that while child health monitoring is globally consolidated around clinical and procedural metrics, there is a systemic neglect of relational and interpersonal dimensions of care. The evidence suggests that current evaluation frameworks are predominantly descriptive and fragmented, often failing to integrate with national health information systems. Regarding future recommendations, it is essential to prioritize the development and validation of standardized relational indicators that capture the patient–provider bond. Furthermore, future research must shift toward longitudinal designs to establish the causal impact of these indicators on long-term health outcomes. Finally, policy-makers should focus on the digital integration of these metrics to ensure that quality assessment in primary health care is not only technically robust but also person-centered and capable of reducing global health inequities.

## Figures and Tables

**Table 1:** Systematization of Well-Child Care Indicators from Studies Included in the Review.

Components	References
Structural Block	
Availability and quality of infrastructure (n = 11)	Al Rashidi et al., 2020 [17]; Brum et al., 2023 [26]; Esamai et al., 2023 [32]; Ezran et al., 2019 [33]; Haskins et al., 2020 [44]; Junqueira and Duarte, 2012 [46]; Lenzi et al., 2014 [50]; Luciano et al., 2014 [51]; Mansbach et al., 2009 [53]; Santos et al., 2015 [66]; Szilagyi et al., 2004 [71].
Availability and quality of general supplies (n = 10)	Abegunde et al., 2015 [16]; Bie et al., 2016 [24]; Bozic and Bajcetic, 2015 [25]; Lee et al., 2024 [49]; Gubert et al., 2021 [43]; Fernández et al., 2019 [36]; Fort et al., 2011 [37]; Sanine et al., 2021 [64]; Santos et al., 2022 [65]; Verguet et al., 2013 [72].
Availability of referral and regulation systems for access to specialized services (n = 9)	Ancira-Moreno et al., 2022 [18]; Araújo et al., 2017 [19]; Fernandes et al., 2022 [35]; Fort et al., 2011 [37]; Pazó et al., 2017 [58]; Gubert et al., 2021 [43]; Kizito et al., 2018 [47]; Nsimba, 2006 [56]; Stevens et al., 2011 [69].
Planning and organization of service delivery and work processes (n = 14)	Al Rashidi et al., 2020 [17]; Ancira-Moreno et al., 2022 [18]; Bálint et al., 2015 [21]; Barry et al., 2018 [22]; Brum et al., 2023 [26]; El-Ayady et al., 2016 [30]; Ezran et al., 2019 [33]; Falisse et al., 2015 [34]; Garjón-Parra et al., 2008 [38]; Pazó et al., 2017 [58]; Haskins et al., 2020 [44]; Marin et al., 2009 [54]; Silva and Alves, 2019 [68]; WHO, 2013 [76].
Availability and quality of the workforce (n = 14)	Al Rashidi et al., 2020 [17]; Bálint et al., 2015 [21]; Burokiene et al., 2022 [28]; El-Ayady et al., 2016 [30]; Fernandes et al., 2022 [35]; Fort et al., 2011 [37]; Gómez-Dantés et al., 1999 [41]; Pazó et al., 2017 [58]; Marin et al., 2009 [54]; Orueta et al., 2015 [57]; Ramírez-Tirado et al., 2014 [62]; Sanine et al., 2021 [64]; Strobel et al., 2018 [70].
**Service Cluster Block For Mch**	
Availability and quality of prenatal care (n = 13)	Ancira-Moreno et al., 2022 [18]; Buranatrevedh et al., 2016 [27]; Engineer et al., 2016 [31]; Esamai et al., 2023 [32]; Ezran et al., 2019 [33]; Falisse et al., 2015 [34]; Marin et al., 2009 [54]; McKay et al., 2024 [55]; Pham et al., 2016 [59]; Ramírez-Tirado et al., 2014 [62]; Verguet et al., 2013 [72]; WHO, 2013 [76]; Wiles et al., 2019 [75].
Availability and quality of child care (n = 29)	Ancira-Moreno et al., 2022 [18]; Arifeen et al., 2005 [20]; Barry et al., 2016 [23]; Barry et al., 2018 [22]; Brum et al., 2023 [26]; Buranatrevedh et al., 2016 [27]; Doubova et al., 2015 [29]; El-Ayady et al., 2016 [30]; Engineer et al., 2016 [31]; Esamai et al., 2023 [32]; Ezran et al., 2019 [33]; Falisse et al., 2015 [34]; Fernandes et al., 2022 [35]; Flores-Quispe et al., 2024 [12]; Fort et al., 2011 [37]; Garjón-Parra et al., 2008 [38]; Gomes et al., 2011 [40]; Gubert et al., 2021 [43]; Haskins et al., 2020 [44]; Koulidiati et al., 2018 [48]; Marin et al., 2009 [54]; Pham et al., 2016 [59]; Plomondon et al., 2007 [60]; Quattrin et al., 2007 [61]; Ramírez-Tirado et al., 2014 [62]; Sanine et al., 2021 [64]; Verguet et al., 2013 [72]; WHO, 2013 [76]; Wiles et al., 2019 [75].
Infrastructure and good immunization practices (n = 11)	Brum et al., 2023 [26]; Esamai et al., 2023 [32]; Falisse et al., 2015 [34]; Fernandes et al., 2022 [35]; Gubert et al., 2021 [43]; Ramírez-Tirado et al., 2014 [62]; Santos et al., 2022 [65]; Verguet et al., 2013 [72]; Weeks et al., 2000 [73]; Weeks et al.s, 2016 [74]; Wiles et al., 2019 [75].

## Data Availability

The original contributions presented in this study are included in the article/Supplementary Materials. Further inquiries can be directed to the corresponding author.

## Supplementary Materials

The following supporting information can be downloaded at https://www.mdpi.com/article/10.3390/children13030382/s1. Table S1: Study search strategy across electronic databases; Table S2: Summary of studies included in the scoping review.

## Abbreviations

The following abbreviations are used in this manuscript:

PHC	Primary Health Care
SUS	Unified Health System
PAISM	Program for Comprehensive Women’s Health Care
PHPN	Program for Humanization of Prenatal and Childbirth Care
