# Supplementary material for: Quality Assessment Indicators for Well-Child Care in Primary Health Care: A Scoping Review of Global Trends, Standardization, and Dimensions of Care
Source: Children (Basel). 2026 Mar 9;13(3):382. doi: 10.3390/children13030382 (PMC13025468; doi:10.3390/children13030382)
Supplement: Supplementary file 1 [file children-13-00382-s001.zip › Table S1.pdf]

Table S1. Study search strategy across electronic databases.

Database	Search strategy
BVS (WHO IRIS; Index Psicologia; BDENF; BBO; WPRIM; BINACIS; CUMED; MINSAPERÚ; IBECS	(“care, primary health” OR “health care, primary” OR “primary healthcare” OR “healthcare, primary” OR “primary care” OR “care, primary” OR “primary health care”) AND (“quality indicators, health care” OR “quality indicators, healthcare” OR “healthcare quality indicator” OR “healthcare quality indicators” OR “indicator, healthcare quality” OR “indicators, healthcare quality” OR “quality indicator, healthcare” OR “health metrics” OR “health metric” OR “metrics, health” OR “healthcare global trigger tool” OR “global trigger tool, healthcare”) AND (db:("IBECS" OR "WHOLIS" OR "BDENF" OR "BBO" OR "WPRIM" OR "BINACIS" OR "CUMED" OR "MINSAPERU" OR "INDEXPSI"))
LILACS	(“indicadores de calidad en la asistencia sanitária” OR “indicadores de calidad de la atención de salud” OR “indicadores de calidad” OR metas OR “metas de salud” OR “métrica sanitária” OR “indicateurs qualité santé” OR “indicadores de qualidade em assistência à saúde” OR “indicadores da eficiência do sistema de saúde” OR meta OR “metas de saúde” OR “métrica de saúde” OR “métricas de saúde” OR “care, primary health” OR “health care, primary” OR “primary healthcare” OR “healthcare, primary” OR “primary care” OR “care, primary” OR “primary health care” OR “quality indicators, health care” OR “quality indicators, healthcare” OR “healthcare quality indicator” OR “healthcare quality indicators” OR “indicator, healthcare quality” OR “indicators, healthcare quality” OR “quality indicator, healthcare” OR “health metrics” OR “health metric” OR “metrics, health” OR “healthcare global trigger tool” OR “global trigger tool, healthcare”) AND (“atención primaria de salud” OR “asistencia primaria” OR “asistencia primaria de salud” OR “asistencia sanitaria de primer nivel” OR “atención básica” OR “atención primaria” OR “atención sanitaria de primer nivel” OR “primer nivel de asistencia sanitaria” OR “primer nivel de atención” OR “primer nivel de atención de salud” OR “primer nivel de atención sanitaria” OR “primer nivel de la asistencia sanitaria” OR “soins de santé primaires” OR “soins de santé primaire” OR “atenção primária à saúde” OR “atendimento básico” OR “atendimento primário” OR “atendimento primário de saúde” OR “atenção básica” OR “atenção básica à saúde” OR “atenção básica de saúde” OR “atenção primária” OR “atenção primária de saúde” OR “atenção primária em saúde” OR “cuidado de saúde primário” OR “cuidado primário de saúde” OR “cuidados de saúde primários” OR “cuidados primários” OR “cuidados primários à saúde” OR “cuidados primários de saúde” OR “primeiro nível de assistência” OR “primeiro nível de atendimento” OR “primeiro nível de atenção” OR “primeiro

	nível de atenção à saúde" OR "primeiro nível de cuidado" OR "primeiro nível de cuidados") AND (db:("LILACS"))
BDTD	**"**(Todos os campos:"Quality Indicators, Health Care" OR "Quality Indicators, Healthcare" OR "Healthcare Quality Indicator" OR "Healthcare Quality Indicators" OR "Indicator, Healthcare Quality" OR "Indicators, Healthcare Quality" OR "Quality Indicator, Healthcare" OR "Health Metrics" OR "Health Metric" OR "Metrics, Health" OR "Healthcare Global Trigger Tool" OR "Global Trigger Tool, Healthcare" E Todos os campos:"Care, Primary Health" OR "Health Care, Primary" OR "Primary Healthcare" OR "Healthcare, Primary" OR "Primary Care" OR "Care, Primary" OR "Primary Health Care")**"**
Embase	"quality indicators, health care" OR "quality indicators, healthcare" OR "healthcare quality indicator" OR "healthcare quality indicators' OR indicator, healthcare quality' OR indicators, healthcare quality' OR 'quality indicator, healthcare' OR 'health metrics" OR "health metric' OR 'metrics, health' OR "healthcare global trigger tool' OR 'global trigger tool, healthcare' AND "care, primary health' OR health care, primary'lexp OR "health care, primary OR 'primary healthcare/exp OR primary healthcare" OR "healthcare, primary' OR primary care'/exp OR 'primary care' OR 'care, primary' OR 'primary health care/exp OR 'primary health care'
Psycinfo	"Care, Primary Health" OR "Health Care, Primary OR "Primary Healthcare" OR "Healthcare, Primary" OR "Primary Care" OR "Care, Primary" OR "Primary Health Care" AND Any Field: "Quality Indicators, Health Care" OR "Quality Indicators, Healthcare" OR "Healthcare Quality Indicator OR "Healthcare Quality Indicators" OR "Indicator, Healthcare Quality OR "Indicators, Healthcare Quality" OR "Quality Indicator, Healthcare" OR "Health Metrics" OR "Health Metric" OR "Metrics, Health" OR "Healthcare Global Trigger Tool OR Global Trigger Tool, Healthcare"
Web of science	ALL=("Care, Primary Health" OR "Health Care, Primary" OR "Primary Healthcare" OR "Healthcare, Primary" OR "Primary Care" OR "Care, Primary" OR "Primary Health Care") AND ALL=("Quality Indicators, Health Care" OR "Quality Indicators, Healthcare" OR "Healthcare Quality Indicator" OR "Healthcare Quality Indicators" OR "Indicator, Healthcare Quality" OR "Indicators, Healthcare Quality" OR "Quality Indicator, Healthcare" OR "Health Metrics" OR "Health Metric" OR "Metrics, Health" OR "Healthcare Global Trigger Tool" OR "Global Trigger Tool, Healthcare")
Medline/pubmed	("quality indicators health care"[All Fields] OR "quality indicators healthcare"[All Fields] OR "Healthcare Quality Indicator"[All Fields] OR "Healthcare Quality Indicators"[All Fields] OR

	(“quality indicators, health care”[MeSH Terms] OR (“quality”[All Fields] AND “indicators”[All Fields] AND “health”[All Fields] AND “care”[All Fields]) OR “health care quality indicators”[All Fields] OR (“indicator”[All Fields] AND “healthcare”[All Fields] AND “quality”[All Fields])) OR “indicators healthcare quality”[All Fields] OR (“quality indicators, health care”[MeSH Terms] OR (“quality”[All Fields] AND “indicators”[All Fields] AND “health”[All Fields] AND “care”[All Fields]) OR “health care quality indicators”[All Fields] OR (“quality”[All Fields] AND “indicator”[All Fields] AND “healthcare”[All Fields])) OR “Health Metrics”[All Fields] OR “Health Metric”[All Fields] OR “metrics health”[All Fields] OR “Healthcare Global Trigger Tool”[All Fields] OR (“quality indicators, health care”[MeSH Terms] OR (“quality”[All Fields] AND “indicators”[All Fields] AND “health”[All Fields] AND “care”[All Fields]) OR “health care quality indicators”[All Fields] OR (“global”[All Fields] AND “trigger”[All Fields] AND “tool”[All Fields] AND “healthcare”[All Fields]))) AND (“care primary health”[All Fields] OR “health care primary”[All Fields] OR “Primary Healthcare”[All Fields] OR “healthcare primary”[All Fields] OR “Primary Care”[All Fields] OR “care primary”[All Fields] OR “Primary Health Care”[All Fields])”
Google scholar	“Healthcare Quality Indicator” OR “Healthcare Quality Indicators” OR “Health Metrics” OR “Health Metric” OR “Healthcare Global Trigger Tool” AND “Care, Primary Health” OR “Health Care, Primary” OR “Primary Healthcare” OR “Primary Care” OR “Primary Health Care”
