# Supplementary material for: Quality Assessment Indicators for Well-Child Care in Primary Health Care: A Scoping Review of Global Trends, Standardization, and Dimensions of Care
Source: Children (Basel). 2026 Mar 9;13(3):382. doi: 10.3390/children13030382 (PMC13025468; doi:10.3390/children13030382)
Supplement: Supplementary file 1 [file children-13-00382-s001.zip › Tables S2.pdf]

Table S2. Summary of studies included in the scoping review.

Author, year (country)	Objective	Study design	Population (sample)	The study used quality indicators (Yes/No)	Do the quality indicators used already exist? (Yes/No)	If not, have the authors validated them?	Main conclusions
Al Rashidi et al., 2020 (Oman)	To assess the performance of PHC services using key performance indicators to identify potential challenges.	Cross-sectional	Primary health care centers (N=12)	Yes	Yes	---	Ten PHC centers achieved a moderate overall performance (67.01%). High scores were observed in safety, satisfaction, timeliness, and accessibility; however, critical gaps were identified in workload management and clinical outcomes.
Araujo, Costa and Pedraza, 2017 (Brazil)	To describe hospitalizations for primary care-sensitive conditions in children under five years of age.	Cross-sectional	Children under age 5 (N=627)	Yes	Yes	---	High rates of hospitalizations for primary care-sensitive conditions, including acute illnesses, were observed, especially among boys with long hospitalization periods.
Arifeen et al., 2005 (Bangladesh)	To describe the quality of care provided to sick children under age 5 in first-level government health facilities.	Cross-sectional	Children (N=284)	Yes	Yes	---	The results suggest that the AIDPI strategy offers a promising set of interventions to address problems in child health services in Bangladesh.
Bálint et al., 2015 (Hungary)	To investigate parental satisfaction with doctors treating children aged 0 to 7 years.	Cross-sectional	Parents of children aged 0–7 (N=980)	Not	---	---	Parents cited both negative and positive factors. Overall, the results show that parents were quite satisfied with their child's family doctor.
Barry et al., 2018 (Ireland)	To apply a set of explicit prescribing indicators to a national pharmacy claims database (Primary Care Reimbursement Service) to determine the prevalence of potentially inappropriate prescribing for children (PIPc) in primary care.	Cross-sectional	Children under age 16 (N=414,856)	Yes	Yes	---	The study found that potentially inappropriate medication use in children was uncommon. However, it indicated opportunities to improve adherence to asthma prescribing guidelines.

Brum et al., 2023 (Brazil)	To assess, from the perspective of users of basic health units, the quality of care for children under two years of age.	Cross-sectional	Users with children under age 2 (N=15,745)	Yes	No	No	Only 36.8% of users reported receiving good quality care for their children, with prevalence decreasing as the child's age increased.
Doubova et al., 2015 (Mexico)	To develop quality-of-care indicators to assess services provided to children with upper respiratory infections at the primary care level and to evaluate such care in family medicine clinics.	Cross-sectional	Children (N=10,677)	Yes	No	Yes	Highlights electronic records as a tool for quality assessment. Identifies a need for strategies to improve evidence-based management of respiratory infections through professional training.
El-Ayady et al., 2016 (Egypt)	To assess adherence and attitudes of primary health care physicians toward the Integrated Management of Childhood Illness guidelines after 17 years of application.	Cross-sectional	Doctors (N=100) Children (N=125)	Yes	Yes	---	Although physicians still showed certain areas of low adherence, those who had used the guidelines since the beginning adhered well to them.
Falisse et al., 2015 (Burundi)	To assess whether performance-based financing contributed to improving indicators related to reproductive health service use in Burundi.	Cross-sectional	Performance-based financing in health services	Yes	Yes	---	Evidence that the Family Development Program improved reproductive health service indicators was weak.
Flores-Quispe et al., 2024 (Brazil)	To assess temporal trends in the quality of health care for children during the first-week consultation at the PHC level.	Cross-sectional	Users of basic health units (N=320,469):<br><br>Women with children under 2 years (N=35,840)	Yes	Yes	---	High-quality consultations during the first week of life increased significantly over a six-year period (from 47.9% to 53.3%). The most substantial improvements were observed in socioeconomically vulnerable regions (low HDI) and areas with full Primary Health Care (PHC) coverage, indicating a reduction in health inequalities.
Gomes, 2011 (Brazil)	To evaluate the quality of healthcare provided to	Cross-sectional	Families of 71 children	No	---	---	Revealed low rates of adequate growth and development monitoring (36.6% in the first year; 28.6% in the second). While prophylactic

	children with sickle cell disease by PHC services in a high-prevalence region.		with sickle cell disease				medication adherence was high (87.3%), significant gaps were identified in vaccination schedules and family knowledge. Caregivers perceived accessibility to health services as poor.
Gubert et al., 2021 (Brazil)	To compare assessments of child health by users of health services participating in PMAQ-AB in Northeast Brazil.	Cross-sectional	Users (N=5,116) of 4,190 Family Health Teams in nine Northeast states.	No	---	---	Indicators with the highest proportions included the Guthrie PKU test within seven days of life (84.4%), continuity of care by the same professionals (79.1%), monitoring of expected growth and development (84.9%), dietary guidance (86.3%), and up-to-date vaccination (95.3%). Differences in child health care quality were observed across states.
Jansson et al., 1998 (Sweden)	To assess the views of mothers and public health nurses concerning a CHP program and the first home visit to parents of newborns.<br>To compare mothers' and nurses' views of CHP programs.	Cross-sectional	Mothers<br>(N=850)<br>Public health nurses<br>(N=291)	No	---	---	Mothers' and nurses' views of quality child healthcare were consistent with official child health promotion goals.<br>Important indicators included kind treatment, competence, adequate time, support, a holistic approach, individualized attention, and home visits to first-time parents.
Junqueira and Duarte, 2012 (Brazil)	To analyze hospitalization rates due to ambulatory care-sensitive conditions (ACSC).	Cross-sectional	Hospitalizations (N=157,003)	No	---	---	Ambulatory Care Sensitive Conditions (ACSC) accounted for 20% of hospital admissions. The most frequent conditions included gastroenteritis, heart failure, and urinary tract infections. A U-shaped trend was identified, with higher hospitalization rates among infants, followed by a decrease in childhood and a gradual increase in older age groups.
Kizito et al., 2018 (Uganda)	To assess the quality of routine childhood tuberculosis (TB) evaluation in Kampala, Uganda.	Cross-sectional	Children (N=11,614)	Yes	No	No	Evaluated the tuberculosis (TB) screening cascade: 47% of children were screened, but only 21% of symptomatic cases underwent diagnostic testing. While 80% of bacteriologically confirmed cases initiated treatment, a critical gap was identified for children meeting clinical criteria, none of whom started treatment.
Koulidiati et al., 2018 (Burkina Faso)	To estimate both crude and effective curative health service coverage provided by rural health facilities to children under 5-years of age in Burkina Faso.	Cross-sectional	Child health providers (N=1,298); clinical cases (N=1,681); Children under 5 years (N=12,497)	Yes	Yes	---	Demonstrated a significant gap between crude coverage (69.5%) and effective coverage. While most facilities showed intermediate quality, only 12.7% were classified as high-quality. Consequently, only 5.3% of children received high-quality services, highlighting that physical access does not guarantee clinical quality.

Lenzi et al., 2014 (Italy)	To examine empirically the pediatric gastroenteritis admission rate indicator in Italy.	Cross-sectional	Pediatric hospitalizations (N=105,890)	Yes	Not	No	Hospitalization risk for non-bacterial gastroenteritis was 24 times higher in infants compared to adolescents, decreasing significantly with age. Nationally, bacterial gastroenteritis admissions were less frequent, but significant regional variability in diagnostic coding was identified, particularly regarding dehydration-related admissions, affecting the indicator's reliability.
Marin et al., 2009 (Argentina)	To evaluate the quality of care provided by primary care health centers (PCHCs).	Cross-sectional	PCHCs (N=338)	No	---	---	Major deficiencies were found in care structure, processes, and outcomes, with health teams lacking key population data. Only 13% of the population used PCHC services, resulting in low vaccination coverage (63.6%), limited child health surveillance (38.8%), and poor early pregnancy detection (6.9%).
Nsimba, 2006 (Tanzania)	To assess prescribing and patient care indicators for children under 5 years with malaria and other conditions.	Cross-sectional	Mothers/guardians with prescriptions (N=652); Sick children under 5 years	Yes	Yes	---	While prescribing quality was high regarding generic use (87%) and adherence to the Essential Drugs List (93.5%), significant failures were identified in the dispensing process. Only 54.7% of drugs were dispensed, and merely 37.2% of caregivers knew how to administer them correctly, highlighting a major gap in pharmaceutical counseling and health literacy.
Orueta et al., 2015 (Spain)	To describe the main process and outcome indicators of primary healthcare services.	Cross-sectional	Children (N=247,493)	No	---	---	After adjusting for patient, doctor, health center and district characteristics, variance in the indicators was mainly attributable to differences among patients, independent of health professional, center, or organization.
Pham, 2016 (Sudan)	To describe the process of using the lot quality assurance sampling (LQAS) method in a conflict-affected region to evaluate PHC program effectiveness.	Cross-sectional	1,323	Yes	Yes: UNICEF's Multiple Indicator Cluster Survey 4 (MICS4) questionnaire	---	From the first to last assessment periods: tetanus toxoid vaccination among pregnant women increased from 47.2% to 69.7%; births attended by a skilled health professional increased from 35.7% to 52.7%; measles vaccinations declined from 72.0% to 54.1%.
Santos et al., 2015 (Brazil)	To characterize hospitalizations of children under 5 years due to primary care-sensitive conditions in Cuiabá, Mato Grosso.	Cross-sectional	Hospitalizations for ACSC (N=16,156)	Yes	Yes	--	Of total hospitalizations, 6,258 (38.7%) were for ACSC and 9,898 (61.3%) for non-ACSC. Although hospitalizations due to ACSC declined, results may indicate deficiencies in the quality of care in the municipality.
Silva and Alves, 2019 (Brazil)	To assess the degree of implementation of PHC attributes as indicators of quality in child care.	Cross-sectional	Child care providers (N=707); Physicians and nurses in	Yes	Yes	---	Professionals rated PHC services higher than users. For professionals, the highest-scoring components were "information systems" and "family guidance" (both 8.9). For users, the best-rated components were "information systems" (7.8) and "utilization" (6.8), while the lowest were "integration of care" (4.0) and "available services" (4.6). In rural

			primary care (N=22)				areas, users rated essential and general service attributes higher than in urban areas, unlike professionals.
Stevens, Vane and Cousineau, 2011 (USA)	To examine whether patient-reported indicators of a quality medical home are associated with health measures among Latino children in low-income families.	Cross-sectional	Children (N=3,258)	No	---	---	Demonstrated a strong positive association between "Medical Home" scores and better Pediatric Quality of Life (total and all subdomains). Higher quality of care was also linked to clinical and functional benefits, including fewer school absences due to illness and significantly improved academic performance in reading and mathematics.
Strobel et al., 2018 (Australia)	To determine associations between social and emotional well-being, anemia, and child neurodevelopment care process indicators.	Cross-sectional	Child health audits (N=1,554)	No	---	---	Children aged 12–23 months were significantly more likely than those aged 24–59 months to receive all process-of-care indicators (PoCIs). For each one-point increase in assessment scores for team structure and function (OR=1.14, 95% CI: 1.01–1.27) and care planning (OR=1.14, 95% CI: 1.01–1.29), the odds of a child receiving an anemia PoCI increased by 14%.
Weeks et al., 2000 (Kyrgyzstan)	To describe a health intervention aimed at improving immunization services in Kyrgyzstan.	Cross-sectional	Health professionals and supervisory structures for immunization services	Yes	Yes	---	The MOH implemented a revised HMIS nationally. Data quality improved, contraindication rates for DPT immunization were reduced to ≤5%, and vaccine wastage decreased substantially. Training health workers in basic epidemiologic skills improved monitoring, enhanced data quality, and fostered worker pride.
Weeks, Ventelou and Paraponaris, 2016 (France)	To identify admissions for outpatient-sensitive conditions, calculate their costs, and measure geographic variation.	Cross-sectional	Hospital discharges (2009: N=1,585,413; 2010: N=1,635,047)	Yes	No	Yes	Reported an upward trend in Ambulatory Care Sensitive Conditions (ACSC) admissions (2009–2010), with higher rates associated with lower income and greater hospital bed availability. The economic impact was significant, exceeding €5 billion annually and accounting for over 8.7 million bed-days, with higher admission rates in France compared to other countries.
Fort et al., 2011 (Guatemala)	To describe a PHC model designed for Guatemala and present evaluation results from 2005–2009.	Repeated cross-sectional	Families (N=3,002); Children (N=3,090); Women (N=8,353)	Yes	Yes	---	Coverage, quality of care, and utilization increased significantly during the 5-year implementation period, suggesting that the model may benefit both outcomes and processes.
Ancira-Moreno et al., 2022 (Mexico)	To develop a set of indicators to assess the quality of maternal and	Validation study	---	Yes	No	Yes	Twenty-two indicators were constructed, 16 of which were selected to assess maternal and child nutritional care at PHC. The systematic use of these 16 indicators in PHC can contribute to improving nutritional status.

	child nutritional care at PHC.						
Barry et al., 2016 (Ireland and the United Kingdom)	To develop a set of prescribing indicators that can be applied to prescribing or dispensing datasets to determine the prevalence of potentially inappropriate prescribing in children in primary care.	Validation study	Specialists (N=15, including general practitioners, pediatricians and pharmacists)	Yes	No	Yes	This study provides a set of 12 evidence-based explicit prescribing indicators to identify potentially inappropriate prescribing in primary care. The list consisted of 12 indicators categorized by respiratory system (n=6), gastrointestinal system (n=2), neurological system (n=2), and dermatological system (n=2).
Garjón Parra et al., 1999 (Mexico)	To select and validate indicators for assessing prescriptions by primary care pediatricians.	Validation study	Specialists – clinicians, pediatricians and pharmacists (N=13)	Yes	No	Yes	The process generated a set of pediatric prescription quality indicators, agreed upon by a representative group of stakeholders and validated for all.
Luciano, 2014 (Italy)	To analyze the validity of the "Pediatric Asthma Hospitalization Rate."	Validation study	Hospitalizations (N=14,389)	Yes	Yes	---	In children aged 2–4 years, the risk of hospitalization for asthma was 14 times higher than in adolescents. Asthma and bronchitis were equally represented as causes of hospitalization and had similar seasonality in preschool children. Patterns of pediatric asthma hospitalization in Italy showed that at least two indicators are needed to measure quality of care accurately.
Mangione-Smith, Schiff and Dougherty, 2011 (USA)	To describe the process used to identify the recommended core set of quality measures as mandated by the Children's Health Insurance Program Reauthorization Act of 2009 (CHIPRA).	Validation study	---	Yes	Not	Yes	- A core set of 25 pediatric quality measures was recommended from 119 candidates, covering preventive care (12), acute care (5), chronic conditions (5), and family experience (2), based on validity, feasibility, and importance.
McKay et al., 2024 (Kosovo)	A criterion validity study of maternal reports of breastfeeding counseling behaviors in primary health facilities.	Validation study	Public PCHCs (N=19); Women with children under 24 months	Yes	No	Yes	Mothers with young children who visited primary care facilities provided valid responses about breastfeeding counseling they received, although more subjective indicators were less reliable.

Wiles et al., 2019 (Australia)	To develop and validate preventive care quality indicators and apply them in general practice.	Validation study	Medical records of children (N=976) from 80 practices	Yes	No	Yes	Developed and validated 43 quality indicators (QIs) and eight care bundles. Clinical practice showed moderate overall compliance (43.3%), with significant variations between domains: immunization reached the highest adherence (80.1%), while specialized physical examinations (visual and musculoskeletal) showed the lowest performance.
Bie et al., 2016 (Netherlands, United Kingdom and Italy)	To describe patterns of outpatient antibiotic use in three European countries, including two new pediatric-specific quality indicators (QIs).	Retrospective cohort	Children (---)	Yes	Yes	---	Antibiotic prescribing patterns varied significantly by age and country. Child-specific QIs, combined with overall prevalence rates, provide a clear picture of trends in community antibiotic prescribing in childhood, allowing the impact of policy interventions to be monitored.
Bozic and Bajcetic, 2015 (Serbia)	To evaluate antibiotic use among the outpatient pediatric population in Serbia using internationally developed disease-specific quality indicators and national guidelines.	Retrospective cohort	Children (---)	Yes	Yes	---	The policy of appropriate antibiotic use has not been effectively implemented in the pediatric population. Prescribing antibiotics without clear indications and the frequent use of broad-spectrum antibiotics remain serious problems.
Buranatreved h et al., 2016 (Thailand)	To examine the quality of health services in primary care units.	Retrospective cohort	Children under age 5 (N=1,440)	Yes	Yes	---	In 2014, primary care consultations in all health centers were below the national standard. Almost all centers reported problems of low birth weight, underweight, and overweight among children under age 5.
Burokiene et al., 2021 (Lithuania)	To review the system of primary health care for children in Lithuania, compare indicators of primary care provided by family doctors and pediatricians, and identify parents' perceptions of the model.	Retrospective cohort	Adults residing in Lithuania (N=1,000)	No	---	---	The results showed a reduction in the number of pediatricians in primary care. Pediatric primary care is more common than care by family doctors and parents tend to trust pediatricians more. The study also showed differences in service models and standards across Lithuanian regions.
Plomondon, 2007 (USA)	To examine the association between primary care provider turnover in managed care organizations and measures of member	Retrospective cohort	Managed care organizations (N=615)	No	---	---	The median provider turnover rate was 7.1% (range, 0%-53.3%). After adjustment for plan characteristics, higher provider turnover was significantly associated with lower member satisfaction and reduced preventive care, including childhood immunization (p=0.045), well-child visits (p=0.002), cholesterol screening after cardiac events (p=0.042), and cervical cancer screening (p=0.024).

	satisfaction and preventive care.						
Ezran et al., 2019 (Madagascar)	To examine changes in maternal and child care content as an indicator of health care quality.	Prospective cohort	Women (N=3,220); Children (N=2,678)	Yes	Yes	---	Over the two-year study period, prescribing rates for sick children and all WHO-recommended perinatal care outcomes increased substantially in the intervention group, with more modest changes in the non-intervention group.
Lee et al., 2024 (Singapore)	To address primary care antibiotic prescription practices for children and benchmark practices against international standards.	Prospective cohort	Children who visited six public primary care clinics	Yes	Yes	---	The study showed the prevalence and appropriateness of antibiotic prescriptions for children within public primary care in Singapore. Gaps were identified in antibiotic prescribing for otitis media and in antibiotic selection for ARI, otitis media, and gastrointestinal conditions.
Mansbach et al., 2009 (USA)	To examine factors contributing to Emergency Department length of stay (ED LOS).	Prospective cohort	Children (N=1,416)	No	---	---	The median ED LOS was 3.3 hours. Longer ED LOS was associated with higher visit volume, Hispanic ethnicity, lack of a primary care provider, night-time presentation, lower oxygen saturation, and hospital admission. Shorter symptom duration and certain clinical interventions were linked to shorter stays.
Szilagyi et al., 2004 (USA)	To measure the impact of the New York State Children's Health Insurance Program on access, utilization, and service quality for enrolled children.	Prospective cohort	Children (2,290); Parents(N=2, 644)	No	---	---	Demonstrated a significant increase in the use of a Usual Source of Care (USC), rising from 47% to 89%. This improved continuity of care was associated with better ratings across multiple quality indicators, including physician-patient interaction and overall care satisfaction, leading to a reduction in parental concerns regarding child health.
Pazó et al., 2017 (Brazil)	To describe time trends in hospitalizations for conditions sensitive to primary care (HCSPC).	Ecological study	Municipalities of the State of Espirito Santo, Brazil (N=78)	No	---	---	Hospitalizations for primary care sensitive conditions decreased by 28.79%, with the highest burden remaining in children under 5 and the elderly. Lower hospitalization rates were significantly associated with expanded Family Health Strategy coverage and a higher density of physicians. Conversely, higher urbanization, income inequality (Gini index), and hospital bed availability were linked to increased admission rates.
Ramírez-Tirado, Tirado-Gómez and López-Cervantes, 2014 (Mexico)	To analyze indicators associated with maternal and infant mortality (<1 year) and assess coverage and variability among Mexican federal entities.	Ecological study	Children Women	Yes	Yes	---	National coverage for maternal and child health indicators was high, ranging from 86.5% to 97.5%. Measles vaccination showed the best performance, while tetanus (TT) vaccination in pregnant women and early breastfeeding initiation were identified as the main gaps. Variations between socioeconomic quintis were noted, particularly in obstetric care and postpartum practices.

Santos et al., 2022 (Brazil)	To identify associations between the structure of basic health units, primary care team processes, social determinants, and hospitalization rates for children under 5 years due to ACSC.	Ecological study	Hospital Information System data, Brazilian demographic census, municipal stratification, and three PMAQ-AB cycles.	No	---	---	Established a link between PHC facility characteristics and hospitalizations for sensitive conditions (ACSC) in children. The study identified specific structural and procedural indicators, alongside municipal socioeconomic factors, as key drivers of hospitalization rates, suggesting that improving facility-level quality and addressing social determinants are essential for reducing avoidable admissions.
Verguet et al., 2013 (South Africa)	To analyze associations between the “Supplementary Immunization Activity 2010” campaign and changes in service use.	Ecological study	District-level monthly headcount data for 52 districts	Yes	Yes	---	A significant decrease was found across eight indicators. Fully immunized children before age 1 decreased by 29% (95% CI: 23–35%, $p<0.001$) during SIA implementation. Contraceptive use and antenatal visits decreased by 7–17% ($p\leq0.02$) and approximately 10% ($p<0.001$), respectively.
Engineer et al., 2016 (Afghanistan)	To test whether pay-for-performance can improve maternal and child services.	Intervention (Cluster randomized trial)	Primary care units (N=442); Households (N=6,908); Married women (N=8,162); Children (N=7,821)	Yes	Yes	---	For pay-for-performance to continue as an approach to improving maternal and child health services, beneficiaries must understand and benefit from their bonuses. However, pay-for-performance does not replace the critical need to strengthen leadership and management capacities in health service organizations.
Esamai et al., 2023 (Kenya)	To assess the impact of the Enhanced Health Care (EHC) model on maternal and child health outcomes.	Intervention (Quasi-experimental)	Women of reproductive age (---); Children under 5 years (---)	Yes	Yes	---	The study concluded that the EHC model significantly improved maternal and child health outcomes, with notable increases in prenatal care coverage, skilled delivery rates, and vaccination rates.
Fernandes et al., 2022 (Mozambique)	To assess the impact of Cyclone Idai on maternal and child health services and	Intervention (Quasi-experimental:	Pregnant women (---); Children	Yes	Yes	---	The study demonstrated the negative effects of extreme weather events on women’s and children’s access to essential interventions. It also showed the usefulness of routine data in monitoring health system

	recovery in Sofala and Manica provinces.	time series analysis)	(---)				performance and resilience during and after shocks, which should be prioritized to inform decision-making.
Fernández et al., 2019 (Spain)	To analyze the impact of a quality improvement initiative aimed at reducing unnecessary treatments for infants with bronchiolitis in primary care.	Intervention (Community intervention)	Children (N=6,371)<br><br>Health centers (N=20)	No	---	---	The mean number of medications per patient decreased from 1.81 (SD: 0.86) to 1.62 (SD: 0.81) ($p<0.01$). Unnecessary treatments for acute bronchiolitis in primary care were significantly reduced. This quality improvement initiative can be implemented in health care centers treating most children with acute bronchiolitis in Western countries.
Haskins et al., 2020 (South Africa)	To evaluate a rapid, scalable, quality improvement (QI) intervention to integrate maternal and child health and HIV services at the PHC level.	Intervention study	Children (N=305) and mothers from 27 clinics	No	---	---	The intervention significantly improved pediatric monitoring quality compared to control clinics. Key improvements included higher rates of anthropometric measurement (77% vs. 63%), more frequent inquiries about infant feeding (74% vs. 67%), and a 14% increase in medical record completeness. The intervention also enhanced maternal health counseling and family planning discussions.
Gómez-Dantés et al., 1999 (Mexico)	To evaluate a program for the non-insured population in the four poorest states of Mexico from 1991–1995.	Program evaluation	---	No	---	---	The program had a positive impact on coverage, accessibility, service quality, and health conditions.<br>However, these changes could not be attributed solely to the program, but to several concurrent activities.
Abegunde et al., 2015 (Nigeria)	To use the continuum of care framework to evaluate the progress of Sokoto State toward achieving MDGs 4 and 5 by 2015.	Program evaluation	Households (N=437)	Yes	Yes	---	Coverage levels of indicators measuring maternal and child care in Sokoto State, Nigeria, fell critically short of the levels recommended to achieve targets 4 and 5, which aimed to reduce maternal and child mortality by one-third by 2015.
Sanine et al., 2021 (Brazil)	To assess the association between organizational quality of child health care in PHC services and management context variables.	Evaluative research	PHC services (151)	Yes	Yes	---	High-quality services were associated with the Family Health Strategy (USF/Mixed) model and outsourced management. Key quality drivers included the permanent availability of family doctors and strong support networks (mental health and childcare services). Surprisingly, participation in national physician provision programs (Mais Médicos/Provab) was not directly associated with quality scores.
Gill, 2014 (United Kingdom)	To improve the quality of care for children and adolescents by defining quality indicators that reflect evidence-based national guidelines and are feasible to audit	Multi-step consensus methodology	National guidelines	Yes	No	Yes	Of 296 national guidelines, 48 were potentially relevant to children in primary care, but only 123 of criteria for translation into 56 potential indicators. Thirteen potential indicators were articulated after review of existing standards.<br>A clinical expert group identified 35 candidate indicators with median scores ≥8 on a 9-point Likert scale, but only 7 achieved a GRADE rating above 1.

	using routine computerized clinical records.						
Gouws et al., 2005 (Brazil, Tanzania and Uganda)	To develop and evaluate summary indices reflecting the quality of care for sick children in first-level facilities.	Index construction and validation study	Children (N=75 in Tanzania; N=240 in Uganda; N=127 in Brazil)	- Index 1: Integrated child assessment.<br>- Index 2: Facility readiness to deliver IMCI.<br>- Index 3: Facility capacity to manage severe illness.<br>- Index 4: Facility capacity to manage illness in young infants.	No	Yes	Experts combined items into inappropriate indicators, producing indices that were difficult to interpret and had limited validity.<br>A revised set of indices, supported by expert and statistical reviews, provided consistent programmatic insights across the three countries.
Sibthorpe et al., 2017 (Australia)	To develop indicators for the diagnosis and treatment of otitis media.	Index construction and validation study	---	Yes	No	Yes	Developed a robust set of seven quality indicators for primary care: six process-oriented (screening, prescribing, care planning, follow-up, referral, and sequelae testing) and one outcome-oriented (disease incidence). The study highlights that indicator selection was constrained by data availability in electronic health records and the extent of primary care's influence over specific health outcomes.
WHO, 2013 (WHO Regional Office for Europe)	To present a tool for assessing the quality of outpatient care during pregnancy and postpartum for women and newborns.	Index construction study	---	Yes	Developed by WHO Regional Office for Europe	No	The tool was designed to assess outpatient prenatal and postpartum care, identify areas for improvement, and inform planning.
Quattrin, 2007 (Italy)	To identify indicators measuring children's social and health status	Documentation research	Sources: the British Paediatric	No	---	---	The selected indicators included neonatal mortality rate, post-neonatal mortality rate, mortality rate for ages 1–14 years, low birth weight,

	most suitable for planning district health services in European countries.		Association, the European Community Health Monitoring Programme and Tamburlini				breastfeeding, vaccine coverage, disability, children covered by protection decrees issued by Juvenile Courts, and abuse/maltreatment. These indicators can serve as a foundation for developing consensus and methods for childhood health care quality assessment at the district level.
Rezapour et al., 2019 (Iran)	To develop a framework for assessing the quality of PHC in the Iranian health system.	Bibliographic review	---	Yes	No	Yes	Developed and validated the Primary Health Care Quality Assessment Framework (PHCQAF) using the Delphi method. The final tool consists of 40 quality indicators (QIs) categorized into seven dimensions: effectiveness (13 QIs), access and equity (8), appropriateness (7), safety (5), governance (3), efficiency (2), and patient-centeredness (2).
